# Emergency Department Visits Among Adolescents for Intentional Assault, Firearm, Poison, and Self-Harm Injuries Before and During the COVID-19 Pandemic in the United States

**DOI:** 10.7759/cureus.69884

**Published:** 2024-09-21

**Authors:** Sidath C Kapukotuwa, Timothy J Grigsby, Jay J Shen

**Affiliations:** 1 Department of Social and Behavioral Health, School of Public Health, University of Nevada, Las Vegas, Las Vegas, USA; 2 Department of Healthcare Administration and Policy, Center for Health Disparities Research, School of Public Health, University of Nevada, Las Vegas, Las Vegas, USA

**Keywords:** adolescent, assault, covid-19, emergency service, firearms, hospital, mental health services, poisoning, self-injurious behavior, socioeconomic factors

## Abstract

Purpose

This study aimed to examine changes in adolescent emergency department (ED) visits for firearm, poison, self-harm, and intentional assault injuries before and during the COVID-19 pandemic and to identify sociodemographic factors influencing these variations.

Methods

Utilizing the Nationwide Emergency Department Sample (NEDS), the study analyzes data for intentional self-harm, poison-related, intentional assaults, and firearm injuries from 2019 to 2021 in adolescents aged 10 to 18 years. A difference-in-differences analysis was conducted to investigate the potential impacts of COVID-19 on injury-associated ED visits, accounting for adjustments related to year, patient factors, and hospital characteristics.

Results

There was a substantial decline in the number of all ED visits from pre-COVID to during the COVID, but we observed increases in the volume of ED visits for firearm-related injuries (13,248 to 20,611; p-value < 0.0001), poison-related injuries (147,812 to 213,753; p-Value < 0.0001), and intentional self-harm-related injuries (153,297 to 229,591; p-value < 0.0001). Conversely, intentional assault-related injuries decreased (167,614 to 154,940; p-value < 0.0001).

During the COVID-19 pandemic, ED visits for intentional self-harm increased by 15% (95% CI = 1.09-1.21), intentional assault by 6% (95% CI = 1.02-1.09), and poison-related injuries by 17% (95% CI = 1.11-1.21) compared to pre-pandemic levels. Racial disparities were observed, with Black individuals having lower odds of self-harm (aOR = 0.63) and poison-related visits (aOR = 0.69) but higher odds of assault-related visits (aOR = 1.40) compared to Whites. Hispanic individuals had fewer ED visits across all injury types, while Native Americans had higher odds across all injury types. Disparities between Black and White individuals in assault-related visits narrowed during the pandemic (aOR = 1.40 to 0.94), but disparities for Native Americans in assault-related injuries widened.

Insurance status influenced ED visits, with Medicaid recipients having higher odds of self-harm (aOR = 1.11) but lower odds of assault visits (aOR = 0.82) compared to those with private insurance. Uninsured adolescents had lower odds of self-harm (aOR = 0.85) and poison-related visits (aOR = 0.95), but higher odds of assault-related visits (aOR = 1.17).

Gender disparities persisted, with females having higher odds of self-harm (aOR = 1.66) and poison-related visits (aOR = 1.42) but lower odds of assault-related visits (aOR = 0.91) compared to males, with these disparities widening for self-harm and poison-related visits during the pandemic.

Conclusions

The findings underscore the heightened vulnerability of adolescents to certain injuries during the COVID-19 pandemic. Targeted interventions and policies to support mental health, firearm safety measures, and strategies to prevent poisoning are recommended. Addressing socioeconomic disparities is necessary to mitigate the impact of intentional injuries among racially/ethnically diverse adolescents during future pandemics.

## Introduction

Deliberate (or intentional) self-harm injuries [1] are prominent contributors to morbidity among adolescents in the United States (US). In 2017 alone, adolescents accounted for 132,000 emergency department (ED) visits due to deliberate self-harm [2]. Notably, adolescents engage in deliberate self-harm at higher rates than any other age group [3], and firearm injury represents the second leading cause of self-inflected death and is a significant source of fatal injuries among American youth [4]. Annually, firearms are responsible for an estimated 5,000 deaths and 22,000 nonfatal injury-related ED visits among American youth [5], and the mean individual hospital cost for a firearm injury is five times greater than that for a motor vehicle injury [6]. Relatedly, assault among individuals aged 10-18 years constitutes 15% of annual deaths and ranks as the third leading cause of death, trailing only unintentional injury and suicide [7]. Despite mortality from violent injury representing only a fraction of the overall burden of violent injuries in adolescents, assault alone contributes to over 200,000 ED visits annually among children aged 10-18 with costs exceeding $15 billion each year [7].

Rates of suicide among 10- to 19-year-olds, which had been on a decline before 2007, experienced a 56% increase from 2007 to 2016. During the same period, rates of suicide among 10- to 14-year-old girls nearly tripled [8]. Among attempted suicides, an important indicator of suicide [9], the estimated ratio of attempted suicides to suicides among adolescents ranges from 50:1 to 100:1 [10]. Data from the 2017 Youth Risk Behavior Survey [11] revealed that 17.2% of high school students seriously considered suicide in a given year, 8.6% self-reported attempting suicide, and 2.2% self-reported receiving medical care for a suicide attempt. Adolescent girls appear to be at an increased risk for suicidal ideation and attempt [12]. From 2000 to 2018, there were 1,627,825 intentional suspected suicide self-poisoning cases, the majority (71%) involving females [9]. Among youth aged 10-15 years, there was an initial decrease in both the number and rate per 100,000 population from 2000 to 2010, followed by a significant increase (125% to 299%) from 2011 to 2018. Of those aged 10-18 years, the increase was predominantly driven by females [9].

The COVID-19 pandemic has significantly impacted adolescent mental health in the US, introducing a complex array of challenges [13]. With disruptions to daily routines, social isolation from peers due to lockdowns and remote learning, increased family stressors, and heightened anxiety about the virus itself, adolescents faced unprecedented mental health pressures. Access to traditional support systems, such as schools and extracurricular activities, became limited, exacerbating feelings of loneliness and disconnection. The pandemic also intensified pre-existing mental health issues and created barriers to accessing professional help, as in-person therapy became more difficult to access for some [14].

Recent data indicate that the percentage of adolescents screening positive for depressive symptoms saw an increase from 5.0% to 6.2%, with more pronounced increases among female, non-Hispanic Black, and non-Hispanic White adolescents [15]. Positive suicide risk screens also rose from 6.1% to 7.1%, and there was a 34% relative increase in the reporting of recent suicidal thoughts among female adolescents. In tandem with the increase in adolescent mental health issues, researchers have documented an increase in externalizing problems, such as intentional and unintentional injury to oneself and others [16].

The COVID-19 pandemic has also intensified pre-existing disparities, including access to care. These inequities are attributed to several factors, including socioeconomic differences, healthcare access, and higher rates of underlying untreated health conditions [17]. Those with inadequate or no health insurance coverage have encountered significant barriers to accessing mental and physical health care resources due to closures resulting from social distancing protocols. Furthermore, the pandemic has led to a significant loss of employer-provided health insurance for many, disproportionately elevating the risk of adverse COVID-19 outcomes among historically marginalized racial and ethnic groups [18].

In 2020, the overall number of pediatric ED visits showed a substantial decline of 51% compared to 2019 [19]. However, the number of visits related to intentional and unintentional injury among youth has evidenced an increase across several studies. Data from the National Syndromic Surveillance Program documented an increase in both the weekly number and proportion of ED visits related to specific types of injuries, including drug poisonings, self-harm, and firearm injuries between 2019 and 2022 [19]. A recent cross-sectional study utilizing the Pediatric Health Information System found an increase in injury-related ED visits at 41 children's hospitals across the US during the pandemic [20]. Furthermore, the results indicated that the mechanisms of injury with the most significant relative increases involved firearms and injuries associated with suicidal intent. Conversely, a nationally representative study of 66 US hospital EDs identified a decrease in nonfatal assault-related ED visits between 2019 and 2020 [21].

This study fills a significant gap in the existing research base [20,21] by assessing changes in ED visits for specific types of injuries among adolescents before and during the COVID-19 pandemic, with a focus on identifying sociodemographic disparities in patterns over time. Furthermore, the use of several major types of injury data than those used in previous studies provides better coverage and generalizable results. The primary objective of this study was to provide a relatively comprehensive picture of the potential impact of the pandemic on major types of injuries among adolescent populations. Specifically, we investigate differences in ED visits among adolescents before and during the COVID-19 pandemic for (a) firearm, poison, and self-harm injuries and (b) intentional assault. The secondary objective was to identify sociodemographic factors associated with observed differences in ED visits across types of injury.

## Materials and methods

Data source and study population

This is a secondary analysis of the Nationwide Emergency Department Sample (NEDS) using ED visits as the unit of analysis. The NEDS is the largest publicly accessible de-identified US ED dataset (about 25-30 million ED visits annually) that represents 20% of stratified all ED visits in the US and encompasses discharge information from ED visits [22]. Weights are available in the dataset from which the national estimate can be used in the analysis. We utilized data spanning from 2019 to 2021 (the latest available to public use), encompassing a total of 6,022,745 ED visits with a weighted national estimate of 25,944,497 ED visits for 10- to 18-year-olds. We selected this age group because many existing studies on adolescent health use the 10-18 age range, ensuring consistency and comparability with other research findings [23]. A data user agreement with the Agency for Healthcare Research and Quality (AHRQ) was completed prior to analysis. Due to the de-identified and publicly accessible nature of this data, the study was reviewed and granted exemption from the institutional review board by the IRB at University of Nevada, Las Vegas.

Measures and statistical analysis

The outcomes of interest were four dichotomously coded variables that were available in the NEDS using the International Classification of Diseases, 10th Edition (ICD-10) codes: intentional self-harm (I10_INTENT_SELF_HARM), poison-related injuries (I10_INJURY_POISON), intentional assaults (I10_INTENT_ASSAULT), and firearm injuries (I10_INJURY_FIREARM) [24]. Please see the supplementary file for the description of the ICD-10 codes (Appendix). These injuries were chosen to capture a broad spectrum of injury types that encompass both intentional and unintentional harm. Furthermore, the selected categories reflect critical areas of concern that have been emphasized in the literature on youth injuries and mental health during the pandemic [19-21]. This approach facilitates a comprehensive analysis of youth injury patterns during the study period, facilitating the identification of trends and associations that might be overlooked if the focus were limited to a single injury.

The primary independent variable was COVID-19 (period), with April-December of 2020 and 2021 representing the period during COVID-19 and 2019 and January-March of 2020 indicating the pre-COVID-19 era. We did not include any earlier years of data because the race/ethnicity information only became available beginning in 2019.

In each weighted multivariable analysis, we specified the independent variable (COVID-19; with the pre-COVID-19 era as the reference year), and covariates including patients' race/ethnicity (Black, Hispanic, Asian, and Pacific Islander, Native American, and White (reference due to their largest volume in the sample)), payer source (Medicaid, uninsured, free care, and private insurance (reference)), median income by ZIP code in quartiles (0-25 percentile, 26-50 percentile, 51-75 percentile, and 76-100 percentile (reference)), age in years, patient residence location (rural (i.e., micropolitan counties, not metropolitan or micropolitan counties), non-rural (i.e., central counties of metro areas of ≥1 million population, "fringe" counties of metro areas of ≥1 million population, counties in metro areas of 250,000-999,999 population, and 4 - counties in metro areas of 50,000-249,999 population) (reference)), and hospital region (Northeast, Midwest, South (reference), and West). Due to a limited sample size of firearm injuries and its very low percentage of the total number of ED visits, we did not include it in the data analysis. Chi-squared test and multiple logistic regression were used for data analysis.

The analysis comprised two primary steps. Initially, descriptive statistics based on the weighted numbers and chi-squared tests were conducted to examine patient characteristics, injury details, and hospital attributes. Subsequently, a difference-in-differences analysis was conducted to investigate the potential impacts of COVID-19 on injury-associated ED visits, accounting for adjustments related to year, patient factors, and hospital characteristics. The observed impact of COVID-19 on disparities (change in disparities in injury) was analyzed through the interaction effects between COVID-19 and covariates, such as race/ethnicity, payer source, and sex. The results are presented using an adjusted odds ratio (aOR) of the interaction term with 95% confidence intervals (95% CI) to assess effects. All statistical analyses were carried out using the SAS including its PROC SURVEYLOGISTIC procedure to account for survey design (SAS 9.4, SAS Institute Inc.), and p-values less than 0.05 (2-tailed) were deemed statistically significant.

## Results

There were 13,102,806 and 12,841,691 ED visits before COVID-19 and during the COVID-19, respectively. The 16-18 age group saw increased visits during COVID-19 compared to before COVID-19, while visits decreased for ages 10-15 except for 12-year-olds. Medicaid was the primary payer source, accounting for over 50% of injury-related ED visits. Rural resident visits slightly increased during COVID-19 compared to before COVID-19, while urban/suburban visits slightly decreased (Table 1). Firearm-related injuries increased during COVID-19 (from 13,248 to 20,611). Poison-related injuries rose from 147,812 to 213,753. Conversely, intentional assault-related injuries decreased from 167,614 to 154,940, while intentional self-harm-related injuries increased from 153,297 to 229,591 (Table 1).

**Table 1:** Injury-related ED visits before and during COVID-19 in the US among adolescents: 2019 to 2021. COVID period = April-December of  2020 and 2021; Pre-COVID period = 2019 and January-March of 2020. Statistical test: chi-squared test.

		Pre COVID-19	During COVID-19	p-value
Patient characteristics				
N		13,102,806	12,841,691	
Age (%)	10	1,078,920 (8.23%)	839,529 (6.54%)	<0.0001
	11	1,073,570 (8.19%)	875,382 (6.82%)	
	12	1,867,084 (14.25%)	1,876,609 (14.61%)	
	13	1,132,581 (8.64%)	1,064,306 (8.29%)	
	14	1,198,132 (9.14%)	1,181,946 (9.20%)	
	15	1,290,857 (9.85%)	1,274,236 (9.92%)	
	16	1,945,137 (14.85%)	2,137,555 (16.65%)	
	17	1,547,519 (11.81%)	1,586,416 (12.35%)	
	18	1,969,006 (15.03%)	2,005,712 (15.62%)	
Sex (%)	Male	6,036,518 (46.07%)	5,833,165 (45.42%)	<0.0001
	Female	7,066,288 (53.93%)	7,008,527 (54.58%)	
Race (%)	White	6,471,098 (49.39%)	6,572,162 (51.18%)	<0.0001
	Black	2,745,016 (20.95%)	2,482,315 (19.33%)	
	Hispanic/Latino	2,943,859 (22.47%)	2,873,970 (22.38%)	
	Asian/Pacific Islander	249,250 (1.90%)	266,857 (2.08%)	
	Native American	81,462 (0.62%)	97,691 (0.76%)	
	Other	612,122 (4.67%)	548,696 (4.27%)	
Insurance status (%)	Medicaid	7,321,207 (55.88%)	7,054,597 (54.94%)	<0.0001
	Uninsured	1,007,247 (7.69%)	810,690 (6.31%)	
	Free care	35,399 (0.27%)	16,436 (0.13%)	
	Private insurance	4,323,949 (33.00%)	4,509,082 (35.11%)	
	Other	415,004 (3.17%)	450,886 (3.51%)	
Location (%)	Urban/Suburban	10,806,041 (82.47%)	10,422,946 (81.16%)	<0.0001
	Rural	2,296,765 (17.53%)	2,418,745 (18.84%)	
Medium Income by ZIP code in quartiles (%)	Lowest quartile	4,621,230 (35.27%)	4,297,508 (33.47%)	<0.0001
	Second quartile	3,529,697 (26.94%)	3,467,834 (27.00%)	
	Third quartile	2,779,799 (21.22%)	2,765,638 (21.54%)	
	Highest quartile	2,172,080 (16.58%)	2,310,711 (17.99%)	
Injury characteristics				
Firearm (%)		13,248 (0.10%)	20,611 (0.16%)	<0.0001
Poison (%)		147,812 (1.13%)	213,753 (1.66%)	<0.0001
Intentional assault (%)		167,614 (1.28%)	154,940 (1.21%)	<0.0001
Intentional self-harm (%)		153,297 (1.17%)	229,591 (1.79%)	<0.0001
Hospital characteristics				
Region (%)	Northeastern	2,399,220 (18.31%)	2,189,176 (17.05%)	<0.0001
	Midwest	2,863,733 (21.86%)	3,312,005 (25.79%)	
	South	5,441,959 (41.53%)	4,892,574 (38.10%)	
	Western	2,397,893 (18.30%)	2,447,936 (19.06%)	

**Table 2:** Sociodemographic factors associated with intentional self-harm (n = 88,270 (382,888 - weighted)), intentional assault (n = 74,948 (322,554- weighted)), and poison (n = 83,925 (361,565- weighted)) associated ED visits before and during the COVID-19. aOR: adjusted odds ratio; CI: confidence interval. * the interaction between COVID and other variables, respectively; with an OR > 1 indicating the odds ratio of a variable versus the reference during COVID was greater than the odd ratio of the variable versus the reference before COVID and an OR < 1 indicating the odds ratio of a variable versus the reference during COVID was smaller than the odds ratio of a variable versus the reference before COVID. Statistical test: weighted multiple logistic regression

	Intentional self-harm			Intentional assault			Poison		
Independent variable	aOR	95% CI	p-value	aOR	95% CI	p-value	aOR	95% CI	p-value
COVID-19 pandemic									
Before COVID (Reference)									
During COVID	1.15	1.09-1.21	< .0001	1.06	1.02-1.09	0.0005	1.17	1.11-1.21	< .0001
Race (before COVID)									
White (Reference)									
Black	0.63	0.61-0.64	< .0001	1.40	1.36-1.42	< .0001	0.69	0.67-0.70	< .0001
Hispanic	0.74	0.72-0.75	< .0001	0.84	0.82-0.85	< .0001	0.80	0.77-0.81	< .0001
Asian and Pacific Islander	1.03	0.98-1.07	0.1437	0.73	0.68-0.76	< .0001	1.04	1.00-1.08	0.0455
Native-American	1.72	1.62-1.82	< .0001	1.41	1.32-1.51	< .0001	1.60	1.50-1.69	< .0001
Change in disparities (During COVID) *									
Black	1.02	0.99-1.04	0.1329	0.94	0.91-0.95	< .0001	1.01	0.98-1.03	0.4304
Hispanic	1.03	1.01-1.05	0.0062	0.99	0.96-1.01	0.3059	1.01	0.98-1.03	0.3806
Asian and Pacific Islander	1.01	0.96-1.04	0.7705	0.97	0.92-1.02	0.2827	0.98	0.94-1.01	0.3093
Native-American	0.99	0.93-1.04	0.6984	1.13	1.06-1.21	0.0002	1.03	0.97-1.09	0.3267
Payer source (before COVID)									
Private Insurance (Reference)									
Medicaid	1.11	1.06-1.16	< .0001	0.82	0.79-0.83	< .0001	1.02	0.97-1.06	0.2985
Uninsured	0.85	0.81-0.89	< .0001	1.17	1.13-1.21	< .0001	0.95	0.90-0.99	0.0325
Free care	0.72	0.61-0.86	0.0003	1.26	1.14-1.40	< .0001	0.77	0.65-0.91	0.0017
Changes in disparities (During COVID) *									
Medicaid	1.03	0.98-1.07	0.2532	0.93	0.91-0.96	< .0001	1.01	0.96-1.05	0.7084
Uninsured	1.00	0.95-1.05	0.9134	1.12	1.08-1.15	< .0001	1.00	0.95-1.05	0.9224
Free care	0.86	0.72-1.02	0.0988	1.11	1.00-1.23	0.0398	0.97	0.82-1.13	0.6912
Sex (before COVID)									
Male (Reference)									
Female	1.66	1.64-1.67	< .0001	0.91	0.90-0.91	< .0001	1.42	1.41-1.42	< .0001
Changes in disparities (During COVID) *									
Female	1.06	1.05-1.06	< .0001	1.07	1.05-1.07	< .0001	1.06	1.05-1.07	< .0001

As shown in Table 2, during COVID-19, the odds of ED visits for intentional self-harm were 15% higher (95% CI = 1.09-1.21), 6% higher (95% CI = 1.02-1.09) for intentional assault, and poison-related injuries were 17% higher (95% CI = 1.11-1.21) compared to pre-COVID-19. Racial disparities varied, with Black individuals having lower odds of self-harm (aOR = 0.63, 95% CI = 0.61-0.64) and poison-related visits (aOR = 0.69, 95% CI = 0.67-0.70) compared to White individuals. However, they had higher odds of intentional assault-related visits (aOR = 1.40, 95% CI = 1.36-1.42). Hispanics exhibited fewer ED visits across all injury types compared to White individuals before COVID-19 and during COVID-19. By contrast, Native American individuals had higher odds across all injury types compared to White individuals before COVID-19 and during COVID-19. Asian and Pacific Islander individuals showed lower odds of intentional assault-related visits (aOR = 0.73, 95% CI = 0.68-0.76) compared to White individuals, but higher odds of poison-related visits (aOR = 1.04, 95% CI = 1.001-1.08).

During the COVID-19 pandemic, racial disparities in ED visits for intentional assaults and self-harm changed compared to before COVID-19. The Black-White disparity in intentional assault-related visits narrowed (aOR = 1.40 to aOR = 0.94), and the Hispanic-White disparity in self-harm slightly narrowed (aOR = 0.74 to aOR = 1.03). Native American disparities in intentional assault-related injuries increased compared to White individuals during the COVID-19 period.

Medicaid recipients had higher odds of intentional self-harm-related visits (aOR = 1.11, 95% CI = 1.06-1.16) but lower odds of intentional assault-related visits (aOR = 0.82, 95% CI = 0.79-0.83) compared to those with private insurance. Uninsured adolescents had lower odds of self-harm (aOR = 0.85, 95% CI = 0.81-0.89) and poison-related visits (aOR = 0.95, 95% CI = 0.90-0.99) but higher odds of assault-related visits (aOR = 1.17, 95% CI = 1.13-1.21) compared to those with private insurance. The same pattern was observed for free care compared to private insurance (Table 2). During the COVID-19 pandemic, payer source disparities in ED visits further increased for international assaults.

Females had higher odds of intentional self-harm (aOR = 1.66, 95% CI = 1.64-1.67) and poison-related visits (aOR = 1.42, 95% CI = 1.406-1.429) but lower odds of intentional assault-related visits (aOR = 0.91, 95% CI = 0.904-0.918) compared to males. These gender disparities widened for self-harm and poison-related visits but narrowed for assault-related visits during the COVID-19 pandemic.

## Discussion

Our findings underscore the importance of a comprehensive approach to understanding youth injuries, particularly during periods of significant societal disruption. By examining a range of injury types, our study provides valuable insights into the complex interplay of factors influencing youth safety and health during the pandemic, contributing to the broader body of research on the public health impacts of the COVID-19 pandemic. These results extend previous findings from single-center studies [25] and compliment trends observed in child [20] and adult populations [26].

The decline in adolescent ED visits observed during COVID-19 compared to 2019 is consistent with previous reports [19,23]. However, the increased number of visits related to firearm, poison-related, and self-harm injuries during the pandemic raises concerns about the mental health and injury risk of adolescents. Previous research has identified significant regional and seasonal differences in suicidal-related injuries among adolescents [23]. These disparities can be attributed to several factors, including socioeconomic status, access to healthcare, availability of trauma centers, and regional variations in public health policies and preventive measures, with peaks during the school year and a notable decline during the spring of 2020 coinciding with COVID-19-related school closures. For example, suicidality was typically higher during the school year, particularly in April and October, and lower during the summer months, particularly in July [23]. Understanding regional and seasonal differences in injury experiences is crucial for developing targeted interventions and policies aimed at reducing injury-related morbidity and mortality among adolescents.

The rise in firearm-related injuries aligns with the overall trend of firearms being a substantial contributor to nonfatal injuries among American youth [4]. Unfortunately, due to a small sample size, we were unable to determine the specific effect of COVID-19 on ED visits related to firearm injuries among adolescents. Nevertheless, these findings underscore the need for firearm safety measures and targeted prevention strategies to reduce adolescent injuries requiring an ED visit. Previous research has identified significant regional and state-level disparities in injury outcomes among adolescents. For instance, a recent study reported notable differences in the incidence and outcomes of adolescent injuries across various regions and states in the US [23]. These disparities can be attributed to several factors, including socioeconomic status, access to healthcare, availability of trauma centers, and regional variations in public health policies and preventive measures. Below, we discuss the sociodemographic patterns and implications for future research and practice.

Intentional self-harm

Native American adolescents had the highest risk of ED visits related to intentional self-harm compared to their White peers. This result is consistent with prior literature that has reported significantly higher rates of self-harm among the Native American population in comparison to the White population [27]. Our results also align with previous literature [27] indicating a lower risk of ED visits related to self-harm among the Black and Hispanic populations. Socioeconomic disparities, cultural attitudes toward mental health services, and the effects of systemic racism and discrimination may play a role [28]. Research has shown that Native American communities face numerous obstacles in accessing mental health services, including being geographically isolated, living in poverty, and a scarcity of care that is culturally sensitive [29]. Cultural perceptions, including the stigma associated with mental health and preference for community-based versus individual approaches to health, significantly influence how various minority groups view and seek mental health support [30]. In addition, chronic stress from facing interpersonal and institutional racism and discrimination can heighten mental distress and lead to an increase in self-harm and suicide rates among these communities [31].

Notably, the Hispanic-White disparities in self-harm were narrowed during the COVID-19 pandemic. This finding appears to contradict previous findings, which have reported a reduction in ED visits related to mental health problems and injuries among Hispanic adolescents during the pandemic [20]. It is important to note that our findings may be specific to self-harm injury-related ED visits among Hispanic adolescents and may not necessarily align with broader patterns in ED visits for mental health reasons or other types of injuries. Furthermore, potential underlying explanations of this observation merit future research. For instance, the worsening of mental health among the youth during the pandemic and the increased reliance on emergency departments as the initial point of contact for mental health care, especially for cases of self-harm as noted by Mitchell et al. (2023) could be significant factors [32]. These issues underscore the impact of the pandemic on mental health services and the accessibility of care, highlighting the need for targeted research to develop strategies to address the mental health needs of vulnerable populations more effectively in future pandemics.

Furthermore, our findings of higher risk of ED visits related to self-harm among females align with previous pre-pandemic literature [27]. Moreover, these higher risks appear to be exacerbated by the COVID-19 pandemic. This may be attributed to more pronounced anxiety, stress, and depression symptoms among adolescent girls during the pandemic [15]. Cross-sectional research across multiple countries has identified gender differences in the psychosocial status of adolescents following the COVID-19 pandemic with girls faring worse than boys [33]. One study noted higher depression scores among girls as a function of perceived worry about contracting COVID-19, disruptions to routine, increased passive social media use, and decreased "in-person” relationships with family and friends [34]. Poor mental health and depressive symptoms specifically are correlated with injury in adolescent samples [35] and may partially explain the gender differences observed in this study. However, further research is recommended to explore gender differences in the association between mental health and injury events.

Our findings demonstrate a significantly higher risk of self-harm injury-related ED visits among adolescents covered by Medicaid and a lower risk associated with free care and uninsured payer sources compared to their counterparts with private insurance, which aligns with previous pre-pandemic analyses [27]. We did not observe any effect of COVID-19 on health insurance status for self-harm injury-related ED visits among adolescents. This is somewhat expected as the ED may serve as a common source of medical care for those without insurance irrespective of extenuating circumstances (e.g., closing of private medical offices to promote social distancing). 

Poison-related injuries

The surge in poison-related injuries is particularly alarming, with a notable overall increase in ED visits from pre-COVID to during COVID-19. Poison-related injuries, whether resulting from drugs or other sources, exhibit almost similar patterns to intentional self-harm-related ED visits for racial and ethnic minority adolescents compared to White adolescents. A previous study conducted before the COVID-19 pandemic showed a similar pattern for non-Hispanic Black and Hispanic adolescents, but they do not report results for Asian and Pacific Islander adolescents [36]. American Indian and Alaska Native (AI/AN) communities face distinctive challenges and health disparities with respect to poison-related injuries. The scarcity of studies specifically focused on poison-related injuries among these groups during the pandemic does not obscure the fact that the AI/AN population has been disproportionately affected by COVID-19 due to a range of systemic health inequities [37].

Furthermore, Williams (2021) underscores the increased COVID-19 mortality rates among Native American individuals compared to other racial groups, a disparity rooted in structural inequalities and racism. Their results show that the standardized mortality ratio for the Native American population not only surpasses that of the White population but also exceeds the ratios for the Black and Latino populations. This suggests an increased vulnerability to poor health outcomes among Native American individuals during the pandemic. Factors exacerbating this vulnerability include socioeconomic conditions, living arrangements, employment in high-risk jobs, and chronic health issues [38] in addition to geographic and infrastructural challenges of reservation life [39].

Finally, the disproportionate rise in poisoning cases among females echoes existing concerns about the mental well-being of adolescents generally [9] and females, specifically, following the COVID-19 pandemic [34]. Prevention efforts should target the specific risk factors contributing to adolescent poisoning, including the identification of substances commonly involved in these events and strategies for restricting access. It is worth noting that due to the nature of the data analyzed in the present study, we cannot conclude whether poisoning visits were due to intentional or unintentional causes, and future research is recommended to uncover patterns in ED visits by poisoning cause (intentional or unintentional) to develop targeted policies and interventions.

Intentional assault

Despite the overall decline in ED visits for intentional assault-related injuries during the pandemic, our findings reveal a significantly higher risk of ED visits related to intentional assault among adolescents during COVID-19. In comparison to White adolescents, Black adolescents had the second-highest risk of ED visits related to intentional assault. Research with adolescents visiting the ED for assault-related injuries has found that retaliatory attitudes were associated with social uncertainty and threat [40], which were high during the pandemic. Moreover, it is critical to consider broader, systemic factors contributing to this elevated risk. Underlying socioeconomic factors, exposure to community violence - a consequence closely linked with socioeconomic status - and systemic issues such as racism and discrimination, create a complex backdrop that increases vulnerability to violence among these groups [41]. These considerations highlight the necessity of adopting a multifaceted approach that goes beyond individual behaviors to address the structural inequalities and societal factors that predispose minority youth to higher risks of violence. During the COVID-19 pandemic, the disparities in intentional assault between Black and White individuals were observed to narrow, a phenomenon that may be attributed to the enhanced role of informal social support networks (extended family members, school personnel, and religious communities) that are widely recognized as crucial protective factors for minority communities [42].

Finally, intersectional factors, including race/ethnicity, income level, and insurance status, intersect to create complex and overlapping vulnerabilities to violence and assault. Policy and structural disparities including systemic racism, socioeconomic inequality, and gaps in social safety nets, contribute to disparities and may influence violence exposure [43]. For example, individuals who belong to multiple marginalized groups, such as Black or Native American adolescents from low-income households with public insurance, may face compounded challenges and increased risk of violence due to intersecting forms of discrimination and systemic inequities. Additional research is needed to identify and intervene in underlying factors related to the pattern of results observed in this analysis.

Furthermore, our findings highlighted a significantly lower risk of adolescent ED visits related to intentional assaults among females compared to males, which aligns with previous literature [44]. However, the advantage of females diminished during the COVID-19 pandemic. This shift suggests that the pandemic exacerbated existing disparities and introduced new dynamics in violence exposure, particularly affecting women. Research with adult samples, for example, noted an increase in domestic violence rates during the pandemic, highlighting the urgent need for enhanced support services for women, including accessible resources for domestic and dating violence, mental health support, and economic assistance during crises [45]. Targeted interventions are essential to mitigate the heightened risks of violence women face during global emergencies [46].

Limitations

The results of this analysis should be interpreted considering several limitations. The constrained sample size hindered a difference-in-differences analysis for firearm injuries, limiting specific conclusions about the impact of the COVID-19 pandemic on related ED visits. To address this, future research integrating multiple datasets or aggregating data from various sources can achieve a larger and more diverse sample overcoming the limitations of this analysis. In addition, these injury categories are not mutually exclusive; an individual injury incident could potentially be classified under more than one category depending on the circumstances (see Appendix). For example, an unintentional firearm discharge might be associated with intentional self-harm or assault behaviors. However, this approach was deliberately chosen to capture the complex and multifaceted nature of injury mechanisms, reflecting the reality of how these incidents often intersect. While the categories are not exhaustive of all possible injury types, they were chosen for their high prevalence and substantial impact on youth morbidity and mortality. This categorization strategy allows us to focus on the most pressing injury issues during the study period, facilitating a more meaningful analysis of trends and patterns. Furthermore, the exclusive focus on ED visits may underestimate the true prevalence of unintentional and intentional injuries among adolescents. To overcome this, researchers could integrate data from other healthcare settings and non-ED visit cases. While the study attempted to establish causality by applying the difference-in-differences approach, caution is warranted for potential unaccounted confounding factors that are not available in NEDS. Future research using a multi-year longitudinal approach to better understand causal relationships is recommended. In addition, the present analysis discusses changes in the number of visits rather than the number of distinct individuals (no patient identifier available) visiting the ED, which implies that repeat visits by the same person for the same or different injuries could potentially inflate the perceived incidence of injuries among adolescents. Novel data collection and management practices that can differentiate between unique individuals and repeat visits with sensitivity to maintaining confidentiality are recommended. Nevertheless, the strength of this study is the use of the NEDS dataset, one of the largest US ED datasets that provides a comprehensive representation of emergency healthcare scenarios. The utilization of the difference-in-differences analysis adds robustness by facilitating comparisons between pre-pandemic and pandemic periods.

## Conclusions

In conclusion, this study offers important insights into the changes in ED visits related to injuries among adolescents before and during the COVID-19 pandemic. The observed racial/ethnic, insurance-based, and gender disparities highlight the need for targeted interventions and policies. These efforts should focus on increasing support for high-risk adolescent populations, including investments in mental health programs aimed at preventing intentional self-harm, strengthening firearm safety measures, and developing strategies to prevent poison-related injuries. 

Future research should further explore these disparities across multiple injury outcomes to better understand their root causes and to develop effective solutions. Notably, motor vehicle accidents, a leading cause of adolescent morbidity and mortality, were excluded from our analysis due to data limitations in the NEDS database. Future studies should consider using databases that capture motor vehicle accident data. Addressing socioeconomic disparities, which were not assessed in this analysis, such as improving access to healthcare through initiatives like Medicaid expansion and equitable treatment options for mental health, may help mitigate the impact of intentional injuries.

## Appendices

Supplementary file 1: description of ICD-10 codes used in the study

I10_INTENT_SELF_HARM

Description: Intent of injury: intentional self-harm [1]

I10_INJURY_POISON

Description: Mechanism of injury: poisoning, including drugs and nondrugs [47]

I10_INTENT_ASSAULT

Description: Intent of injury: assault [48]

I10_INJURY_FIREARM

Description: Mechanism of injury: firearm [49]

**Table 3:** Basic structure of the 2014 proposed ICD–10–CM external cause-of-injury matrix for categorizing mechanism and intent of injury. † Indicates that relevant ICD–10–CM external cause-of-injury codes exist for this cell. … Category not applicable (no relevant ICD–10–CM external cause-of-injury codes exist for this cell). ‡ New mechanism category in the 2014 proposed ICD–10–CM external cause matrix that is not found in the ICD–9–CM external cause matrix. Adapted from: Hedegaard, H., Johnson, R. L., Garnett, M. F., & Thomas, K. E. (2019). The International Classification of Diseases, 10th Revision, Clinical Modification (ICD-10-CM) External Cause-of-injury Framework for Categorizing Mechanism and Intent of Injury. National health statistics reports, (136), 1–22 [50].

Mechanism of injury	Intentional self-harm	Assault
Cut/pierce	†	†
Drowning/submersion	†	†
Fall	†	†
Fire/burn	†	†
Fire/flame	†	†
Hot object/substance	†	†
Firearm	†	†
Machinery	…	…
All transportation^‡^	†	†
Motor vehicle–Traffic (MVT)	†	†
MVT–Occupant	†	†
MVT–Motorcyclist	…	…
MVT–Pedal cyclist	…	…
MVT–Pedestrian	…	†
MVT–Other^‡^	…	…
MVT–Unspecified	…	…
Motor vehicle–Nontraffic^‡^	…	…
Pedal cyclist, other	…	…
Pedestrian, other	…	…
Other land transport	†	†
Other transport	†	†
Natural/environmental	†	†
Bites/stings, nonvenomous^‡^	…	†
Bites/stings, venomous^‡^	†	†
Natural/environmental, other	†	†
Overexertion	…	…
Poisoning	†	†
Drug^‡^	†	†
Nondrug^‡^	†	†
Struck by/against	†	†
Suffocation	†	†
Other specified	†	†
Other specified, child/adult abuse^‡^	…	†
Other specified, foreign body^‡^	…	…
Other specified, classifiable	†	†
Other specified, not elsewhere classifiable	†	†
Unspecified	†	†
